# Smoking and HIV: what are the risks and what harm reduction strategies do we have at our disposal?

**DOI:** 10.1186/s12981-018-0213-z

**Published:** 2018-12-12

**Authors:** Michelle L. Giles, Coral Gartner, Mark A. Boyd

**Affiliations:** 1 Department of Infectious Diseases, Alfred Health, Melbourne, VIC Australia; 2 Department of Obstetrics and Gynaecology, Monash University, Melbourne, VIC Australia; 3 School of Public Health, Faculty of Medicine, The University of Queensland, St Lucia, QLD Australia; 4 Faculty of Health and Medical Sciences, University of Adelaide, Adelaide, SA Australia; 5 Kirby Institute, University of New South Wales, Sydney, NSW Australia; 6 Queensland Alliance for Environmental Health Sciences, Faculty of Health and Behavioural Sciences, The University of Queensland, St Lucia, QLD Australia

**Keywords:** HIV, Smoking, Harm Reduction, Nicotine

## Abstract

The World Health Organization estimates that smoking poses one of the greatest global health risks in the general population. Rates of current smoking among people living with HIV (PLHIV) are 2–3 times that of the general population, which contributes to the higher incidence of non-AIDS-related morbidity and mortality in PLHIV. Given the benefit of smoking cessation, strategies to assist individuals who smoke to quit should be a primary focus in modern HIV care. Tobacco harm reduction focuses on reducing health risk without necessarily requiring abstinence. However, there remains uncertainty about the safety, policy and familiarity of specific approaches, particularly the use of vaporised nicotine products. Evidence suggests that vaporised nicotine products may help smokers stop smoking and are not associated with any serious side-effects. However, there is the need for further safety and efficacy data surrounding interventions to assist quitting in the general population, as well as in PLHIV specifically. In addition, official support for vaping as a harm reduction strategy varies by jurisdiction and this determines whether medical practitioners can prescribe vaporised products and whether patients can access vaporised nicotine products. When caring for PLHIV who smoke, healthcare workers should follow general guidelines to assist with smoking cessation. These include: asking the patient about their smoking status; assessing the patient’s readiness to quit and their nicotine dependence; advising the patient to stop smoking; assisting the patient in their attempt to stop smoking through referral, counselling, pharmacotherapy, self-help resources and/or health education; and arranging follow-up with the patient to evaluate their progress.

## Background

The World Health Organization estimates that smoking poses one of the greatest global health risks in the general population [1]. After high blood pressure, smoking is ranked second among the 10 leading risk factors in the world causing death, and is ranked first in high-income countries [2]. Smoking substantially increases the risk of death from lung and other cancers, heart disease, stroke and respiratory disease. Globally, smoking causes 71% of lung cancers, 42% of chronic respiratory disease, 10% of cardiovascular disease, and is responsible for 12% of male deaths and 6% of female deaths [1]. Smoking is a risk factor that is associated with more than one disease, so focusing efforts on cessation and harm reduction from smoking has the potential to ameliorate multiple causes of disease.

Cardiovascular disease and non-AIDS malignancies have become major causes of death among people living with HIV (PLHIV) [3]. The relative impact of HIV-related factors versus lifestyle factors, such as smoking, on these causes of death is often debated. Many cohort studies have reported higher rates of smoking among PLHIV than the general population [4, 5]. In a nationwide, population-based cohort study, all-cause and non-AIDS-related mortality was reported as higher among smoking compared with non-smoking PLHIV (mortality rate ratio 4.4, 95% confidence interval 3.0–6.7) [4]. In this study from Denmark, where antiretroviral therapy is free and HIV care is well organised, PLHIV who smoked lost more life-years to smoking than to HIV (12.3 years life lost associated with smoking versus 5.1 years life lost associated with HIV status) [4]. The excess mortality of smokers was tripled among those who were HIV-positive, compared to the background population and the population attributable risk of death associated with smoking was 61% among HIV positive patients compared with 34% among controls [4]. Similar impacts on life expectancy among PLHIV have been reported from other European countries and North America [5]. Importantly, those who quit smoking had a 40% lower risk of death compared with current smokers [4].

Compared to the general population, PLHIV have a more than twofold higher incidence of non-AIDS-related morbidity, including cancer and myocardial infarction. In addition PLHIV are at higher risk than non-smokers with HIV of developing bacterial pneumonia, *Pneumocystis jiroveci* pneumonia and COPD [6–9].

The excess in smoking-related morbidity and mortality also increases with age, so given that PLHIV are predicted to have an almost normal life expectancy, smoking-related morbidity and mortality is also likely to increase in this population [5].

Approximately 70% of myocardial infarctions among PLHIV can be attributed to smoking [10]. In addition, the reversibility of excess myocardial infarction risk after stopping smoking begins after 1 year and continues to decline even beyond 3 years after quitting [11].

Approximately 27% of cancers in PLHIV are attributable to smoking [12]. In the absence of smoking, the risk of cancers unrelated to viral infections is not elevated and is similar to other morbidities, and the incidence for both infection- and non-infection-related malignancies increases with age [13]. Unlike the early benefits seen for myocardial infarction risk after quitting smoking, the lung cancer risk remains elevated, with no decline in incidence observed over 7–8 years after stopping in a cohort study of PLHIV [14]. However, in a microsimulation model-based analysis, quitting smoking did eventually impact the risk of lung cancer in people living with HIV, albeit over a lifetime [15].

## Tobacco harm reduction strategies for people living with HIV

Despite data demonstrating the excess number of lives lost due to smoking compared with HIV itself, rates of current smoking among PLHIV remain 2–3 times that of the general population. Given the individual benefit of smoking cessation, strategies to assist individuals who smoke should be a primary focus in modern HIV care. Yet it is often overlooked. Unfortunately, smoking is a difficult addiction to break with reports that 80% of smokers who attempt to quit on their own relapse within 1 month and only 5% achieve long-term abstinence [16]. Tobacco harm reduction strategies are based on the utilization of innovative tobacco products, reduced tobacco consumption and pharmaceutical medication. In a systematic review published in 2016, there was evidence supporting nicotine replacement therapy (albeit assessed as low quality) but a lack of evidence for other harm reduction aids such as behavioural support [17].

Barriers identified to addressing smoking among PLHIV by health practitioners have included lack of confidence in nicotine replacement prescribing, competing priorities, lack of skills or knowledge, uncertainty around referral pathways and lack of confidence in the patient’s ability to quit [18].

Tobacco harm reduction focuses on reducing health risk without necessarily requiring abstinence, similar to the philosophy underpinning public health programs relating to pre-HIV exposure prophylaxis and clean needle and syringe exchanges. Studies have reported that health practitioners generally support tobacco harm reduction strategies and believe that these could benefit PLHIV [18]. However, there remains uncertainty about the safety, policy and familiarity of specific tobacco harm reduction approaches, particularly the use of vaporised nicotine products [18, 19].

In addition to practitioner barriers, there are unique challenges faced by HIV-positive individuals that may impact on attempts to stop smoking such as engagement in HIV care, concurrent substance use and antiretroviral adherence [20].

## Current options for substitution

Long-term substitution has greater potential for substantial health benefit than ‘cutting down’ [21]. There are two approaches to substitution: a therapeutic approach similar to methadone maintenance therapy, and a non-therapeutic approach using unapproved products, including vaporisers. Current options for nicotine replacement include patches, gum, lozenges, mouth sprays and transdermal products. Vaporisers (e-cigarettes) simulate smoking without the smoke and can be used with or without nicotine. Ideally, nicotine replacement therapy treatment has a limited time period with the aim of moving the smoker off nicotine entirely. The legal status of vaporisers, and the nicotine-containing liquid used in them, varies by jurisdiction. In many countries, they are legally sold as consumer products, similar to cigarettes. However, some countries only allow nicotine-free vaporisers and refill fluids to be sold.

A Cochrane review of the effectiveness of e-cigarettes concluded that they may help smokers stop smoking and were not associated with any serious side-effects when used for up to 2 years [22]. This Cochrane review however does report the limitations in the current literature in terms of small numbers of studies, few events and wide confidence intervals in the studies included [22]. In population studies, e-cigarette users (herein referred to as ‘vapers’) were more likely to quit (and succeed in quitting) compared with non-users [23–25]. In addition, long-term vapers were four times more likely to cease smoking compared to non-users, and among those who made a quit attempt, use of e-cigarettes as a cessation aid was more effective than FDA-approved pharmacotherapy. Not all studies however have reported positive findings with e-cigarettes, with some studies reporting no association with lower rates of smoking cessation with short-term use [26]. However, this same study did report long-term use of e-cigarettes associated with higher rates of smoking cessation [26].

The Royal College of Physicians and Public Health England have estimated that the risk of long-term use of e-cigarettes is unlikely to exceed 5% of the harm done as a result of smoking tobacco [27–29]. The National Academies of Sciences, Engineering and Medicine have also concluded that “e-cigarettes appear to pose less risk to an individual than combustible tobacco cigarettes” and that “they might also increase adult cessation of combustible tobacco cigarettes” [21]. However, the committee highlighted the need for more evidence on both short-term and long-term effects of vaping.

Given the high rates of smoking among PLHIV, the well-recognised increased morbidity and mortality in this population attributable to smoking, and the need for safety and efficacy data surrounding interventions to assist quitting, it is encouraging that studies in PLHIV are being published. Two recent examples include: a feasibility study exploring the use and acceptability of vaporised nicotine products among PLHIV [30], with a larger randomised controlled trial commencing recruitment in 2018; and a phase III randomised controlled trial of varenicline reporting a higher proportion of patients achieving abstinence over the study period in the treatment group compared to placebo [31].

## Ethical and policy considerations

The role of tobacco harm reduction strategies, and in particular vaping, is still hotly debated. Those opposed to vaping are concerned about the limited evidence from randomised controlled trials for their efficacy and safety, and the potential for e-cigarettes to introduce new young users to nicotine addiction [32]. Those in favour argue that e-cigarettes have the potential to save lives by assisting smokers to quit, are safer than smoking, and provide a harm reduction strategy that addresses the behavioural aspects of smoking addiction [33].

Official support for vaping as a harm reduction strategy varies by jurisdiction. Health authorities in the United Kingdom support vaping as a smoking cessation and harm reduction method, although they are not approved as medicines. In the United States, the Center for Disease Control state in their information produced on e-cigarettes that they have the potential to benefit adult smokers but also state they have potential for harm [34]. In 2017, the newly appointed FDA Commissioner announced that the agency’s approach to regulating nicotine and tobacco products would take account of the continuum of risk, combining a nicotine reduction approach for combustible tobacco products while allowing innovation in less harmful nicotine products, such as e-cigarettes, so that “adults who still need or want nicotine could get it from alternative and less harmful sources” [35]. E-cigarettes are not currently approved by the FDA as a quit smoking aid and the Centers for Disease Control and Prevention advise that the evidence is insufficient to recommend e-cigarettes for smoking cessation, while acknowledging they may help “adult smokers if used as a complete substitute for all cigarettes and other smoked tobacco products” [34].

It is within this environment that healthcare workers must function, as these policies may determine restrictions in prescribing and influence access for patients.

## Practical suggestions

Below is a list of practical suggestions developed by the authors for healthcare workers to support PLHIV to quit smoking.

1. The 5As: (i) Ask about smoking status; (ii) Assess readiness to quit and nicotine dependence; (iii) Advise patient to quit; (iv) Assist (referral, counselling, pharmacotherapy, self-help resources, health education); and (v) Arrange follow-up to evaluate progress.
2. Consider pharmacotherapy for nicotine addiction. Varenicline and nicotine replacement therapy are the two most efficacious approved treatments. The most reliable indicator of nicotine dependence is the time to first cigarette after waking in the morning (< 30 min).
3. Nicotine replacement products come in slow (i.e. nicotine patches), medium (i.e. nicotine gum) or fast (i.e. mouth sprays) acting preparations. Most people who require nicotine replacement need a combination of these, such as slow-acting to address the background craving and rapid-acting to address the cue-induced cravings.
4. Nicotine patches are more effective if started 2 weeks before the day of quitting and lead to no additional adverse effects.
5. Be armed with the facts; nicotine does not cause cancer and nicotine replacement therapy is always safer than smoking. Smokers should be counselled not to under-dose with nicotine replacement therapy products or discontinue use too early, as this can lead to relapse to smoking.
6. Behavioural counselling involves an assessment of triggers for smoking, barriers to quitting and other related lifestyle changes, such as exercise and alcohol use. Pharmacotherapy is more effective when combined with counselling support.
7. When discussing e-cigarettes, healthcare professionals can provide the following advice: they are a lower-risk alternative to smoking and although there may still be some risks with long-term use, continuing to smoke is much more harmful; they deliver nicotine and a ‘smoking experience’; correct technique is to take longer, slow puffs; daily use is more effective than intermittent use; the aim is to stop smoking completely (ideally within 3–6 months); for safety, use the correct battery charger; and keep e-liquid out of reach of children.

## Abbreviation

PLHIV: people living with HIV
